# The Gittins Index for a Transparent One-Armed Bandit with a Continuous Payoff Spectrum in Equilibrium States

**DOI:** 10.3390/e28080875

**Published:** 2026-08-04

**Authors:** Marcin Makowski, Edward W. Piotrowski, Jan L. Cieśliński

**Affiliations:** 1 Faculty of Physics, Department of Mathematical Methods in Physics, University of Białystok, ul. Ciołkowskiego 1L, 15-245 Białystok, Poland; j.cieslinski@uwb.edu.pl; 2 Independent Researcher, 15-667 Białystok, Poland; qmgames@gmail.com

**Keywords:** Gittins index, optimal stopping, Kelly criterion, Kullback–Leibler divergence, reinforcement learning

## Abstract

We present an analogue of the classical Gittins index for a one-armed decision problem with a continuous spectrum of payoffs. The model assumes that the decision-maker observes independent realizations of a random variable and, at each step, decides whether to accept the current opportunity or continue observing. We show that the optimal strategy takes the form of a threshold rule, while the corresponding reservation index is determined by a one-dimensional fixed-point equation with a direct decision-theoretic interpretation. The model is illustrated with an example of bookmaker betting related to horse racing and the Kelly criterion. This perspective allows the proposed index to be viewed as a threshold of informational advantage. This, in turn, points to potential applications in optimal stopping problems and decision-making under uncertainty.

## 1. Introduction: The Gittins Index

The multi-armed bandit problem is a classical problem in probability theory and machine learning, concerning sequential choice among competing sources of random payoffs. At each step, the decision-maker selects one of the arms, observes the outcome, and updates the subsequent strategy on the basis of this information. The central challenge in this class of models is to find a rule that orders the arms according to their current attractiveness in the simplest possible way. To this end, a one-dimensional index is assigned to each arm. In the classical Bayesian setting, the seminal result of John C. Gittins shows that, for an infinite time horizon and exponential discounting, the strategy of selecting the arm with the largest index is optimal [1,2].

What is the index assigned to an arm in a given state? Its classical definition follows from reducing the multi-armed problem to an auxiliary one-armed stopping problem. It is assumed that the state of a single arm evolves as a Markov process ${\left(S_{t}\right)}_{t\geq 1}$ on a state space *S*, with transition kernel P(x,·), where the initial state is x∈S. Each state is associated with an immediate reward $r(S_{t})$, and future payoffs are discounted by a factor α∈(0,1). If in each round the player may either stop the process or continue activating the arm, then the value of the auxiliary problem can be written as

$$v_{\gamma }\left(x\right):=\underset{\tau \in T}{\sup}E_{x}\;\left[\sum_{t=1}^{\tau -1}\alpha^{t-1}\left(r(S_{t})-\gamma \right)\right],$$

where T denotes the set of stopping times with respect to the natural filtration of the process, taking values in {1,2,…}. The parameter γ plays the role of a continuation cost or an outside value, whereas the choice τ=1 corresponds to immediate stopping, and hence to an empty sum and a value equal to 0. The Gittins index of state *x* is defined as the smallest value of the charge γ at which the decision-maker becomes indifferent between immediate withdrawal and further play; that is,

$$g\left(x\right):=\inf\{\gamma \in R:\;v_{\gamma }\left(x\right)=0\}.$$

Equivalently, this index can be written as

(1) $$g\left(x\right)=\underset{\tau \geq 2}{\sup}\frac{E_{x}\;\left[\sum_{t=1}^{\tau -1}\alpha^{t-1}r\left(S_{t}\right)\right]}{E_{x}\;\left[\sum_{t=1}^{\tau -1}\alpha^{t-1}\right]}.$$

The second form shows that the Gittins index is the maximal discounted average reward attainable by an optimal choice of the stopping time in a one-armed problem [1].

It is worth explicitly explaining all the elements that make up the above definition. By x∈S we denote the fixed current state of the arm from which the analysis of the auxiliary stopping problem begins. This does not mean that the state of the arm remains equal to *x* throughout. On the contrary, the subsequent evolution is described by the process ${\left(S_{t}\right)}_{t\geq 1}$, with *x* serving only as the initial state; i.e., $S_{1}=x$. The Gittins index g(x) is therefore the value assigned to an arm that is currently in state *x*. In Bayesian models, *x* usually takes the form of a concise summary of the observations made so far, containing all information relevant for determining the current posterior distribution. The transition kernel *P* of the Markov process describing the evolution of the arm’s state defines the probability distribution of the state to which the arm moves after the next activation.

The function *r* determines the one-step payoff from activating the arm in a given state. This means that if at time *t* the arm is in state $S_{t}=x$, then the current activation is associated with the reward r(x), which enters the sum of discounted payoffs. It is usually assumed that rewards are random. Then, if $Y_{t}$ denotes the actual random reward received at the *t*-th activation of the arm, and before this activation the arm was in state $S_{t}=x$, the function *r* assigns to this state the expected value of that reward; namely,

$$r\left(x\right)=E\left[Y_{t}|S_{t}=x\right].$$

The parameter α plays the role of a discount factor in the model. The smaller its value, the greater the weight assigned to current payoffs and the smaller the weight assigned to benefits deferred in time. By τ we denote a stopping time; that is, a rule deciding how long it is worth continuing the exploration of a single arm. The parameter γ plays the role of the price of further play with a given arm. It can be viewed as a cost incurred at each decision to continue. The Gittins index is then the threshold price that is still worth paying for the possibility of further play in state *x*.

In the quotient representation of the Gittins index, the numerator

$$E_{x}\;\left[\sum_{t=1}^{\tau -1}\alpha^{t-1}r\left(S_{t}\right)\right]$$

denotes the expected discounted total reward that can be obtained from the present moment until stopping, if the process starts in state *x*. In turn, the denominator

$$E_{x}\;\left[\sum_{t=1}^{\tau -1}\alpha^{t-1}\right]$$

describes the expected discounted length of continuation; that is, the discounted number of future activations of the arm until stopping. The quotient of these two quantities can therefore be interpreted as the expected discounted average reward from further activation of the arm, calculated from the perspective of the current state *x*.

In the multi-armed model, each arm *i* is assigned its current index $g(S_{i}\left(t\right))$, and then the arm with the largest index is selected. In the classical discounted Bayesian model, such a rule is an optimal policy. As a result, the complex comparison of entire future random paths is reduced to a comparison of real numbers assigned to individual arms [1,2].

The Gittins index itself, as well as all kinds of its contemporary analogues and generalizations, are intensively studied tools in broadly understood decision theory. Recent studies have developed methods for computing and approximating the index [3,4] and explored its applications in scheduling and queueing theory [5,6]. Index ideas also appear in Bayesian optimization, including problems of costly exploration and stopping rules [7,8], as well as in reinforcement learning [9]. Contemporary analogues of the Gittins index are also used in graph exploration [10], security games [11], platform models [12], and search problems with travel costs [13]. The paper by Scully and Terenin [14] is of a survey nature, and in it the authors slightly change the perspective on the Gittins index. It is not merely a difficult dynamic programming equation, but a universal principle for designing decision algorithms.

It is worth noting the broad range of potential applications of methods based on Gittins’s idea. They have been applied in IoT systems, where they are used, among others, for scheduling data transmission with unmanned aerial vehicles (UAVs) [15], client selection in so-called decentralized federated learning [16], as well as for reducing the costs of distributed machine learning [17]. Similar ideas can also be found in other areas of scientific research, such as adaptive clinical trial design [18], the theory of reservation prices in optimal stopping problems [19], knowledge-gradient methods, and preference learning [20,21]. Taken together, these examples provide a strong motivation for studying the multi-armed bandit problem.

Although the proposed value is referred to as a “Gittins index analogue,” we must explicitly highlight a fundamental difference from the classical framework. Because our model relies on independent and identically distributed observations without state evolution, the classical exploration–exploitation dilemma is absent. The analogy to Gittins’s work lies strictly in the structural methodology of the solution: as in the classical approach, we reduce the multi-armed selection process to an auxiliary one-armed optimal stopping problem. We define the index as a reservation threshold representing the point of indifference, and demonstrate that the multi-armed choice simplifies to picking the arm with the highest index. In this sense, our approach adopts the index-based decision architecture of Gittins’s original idea, adapting it to a transparent, stationary setting.

## 2. Motivation and Outline of the Paper

The aim of this paper is to propose a transparent analogue of the classical Gittins index approach for a one-armed decision problem with a continuous spectrum of payoffs. This article refers to the intuition underlying the multi-armed bandit problem, but formulates it in a more transparent model. Unlike the standard Markov model with discounted reward, we consider a model based on a sequence of independent observations and the criterion of average payoff per cycle. These observations may be interpreted in various ways. Since in the article we primarily refer to a market analogy, we shall interpret them as the potential net profit from a transaction, the rate of return from an opportunity, or, more broadly, the payoff associated with a given opportunity. Acceptance means realizing this opportunity and obtaining the payoff, whereas rejection means moving on to the next observation. After entering into a transaction, there is a deterministic time equal to 1, interpreted as the time of execution, holding, or settlement of the position. This assumption reflects a natural market constraint on capital liquidity: funds committed to one transaction cannot be used immediately to exploit another opportunity, and the execution and settlement of the position itself require time.

Analogously to the classical Gittins idea, the attractiveness of a given investment opportunity is assessed by analyzing an auxiliary one-armed problem; that is, a problem associated with a single source of offers. The number obtained in this way is then used to compare different sources of market opportunities. As in the case of the Gittins index, this number has the interpretation of a boundary value, the so-called reservation threshold. It is the value of the current investment opportunity at which the investor regards immediate acceptance and continued waiting as equally beneficial. In other words, this threshold determines the boundary between a situation in which it is still worthwhile to wait for a better opportunity and a situation in which the available transaction should already be realized. In this sense, it determines the minimal attractiveness of an opportunity at which entering into the transaction becomes optimal.

The structure of the problem under consideration appears in a broad class of sequential decisions. Its characteristic elements are the random arrival of successive opportunities, an immediate and irreversible decision to accept or reject the current opportunity, the time cost associated with further search, and a period during which the resource is unavailable after an offer is accepted. In such situations, the threshold $r^{⋆}$ determines the minimum value of the current opportunity that compensates for giving up further search.

A particularly natural interpretation comes from optimal foraging theory. A predator or consumer encounters successive potential food sources with random energy values. Rejecting an item means continuing the search, while accepting it yields energy and requires additional time for capture, preparation, or consumption. Classical models of diet choice and attack strategies maximize the long-run rate of energy intake and lead to rules determining which encountered opportunities should be accepted [22,23,24]. In this interpretation, the symbol $X_{n}$ used later in the paper may denote the net value of the *n*-th encountered food source, and the optimal threshold $r^{⋆}$ is the minimum value that justifies its acceptance.

A similar structure appears in psychological studies of sequential choice and search. Unlike standard experiments in which all options are presented simultaneously, in foraging tasks successive opportunities become available one at a time. The decision-maker must then compare the current benefit with the opportunity cost of the time required for further search. Studies of learning in such tasks indicate that the long-run average reward rate may serve as an adaptive reference level [25]. The threshold rule is also related to the concept of satisficing, according to which the search ends when the first opportunity exceeding a fixed aspiration level is found [26]. In the model presented here, however, the aspiration level is not chosen arbitrarily, but results from the solution of the fixed-point equation and maximizes the average reward per unit time.

From an algorithmic perspective, the model is related to sequential selection problems. In these problems, values are revealed successively and the decision-maker must immediately accept or reject each one. Threshold rules play an important role, among other areas, in prophet inequalities and posted price mechanisms [27,28]. Unlike fixed-horizon models, the process considered here renews after each acceptance, and the optimality criterion is the average reward per unit time. The model can therefore also be interpreted as a simplified admission control problem: successively arriving tasks have random values, a rejected task does not occupy the resource, while an accepted task blocks it for a fixed service time.

The construction presented here is not intended to reproduce the full complexity of each of the applications mentioned above, but should be treated as a deliberately simplified baseline model. Temporal dependence, non-stationarity, random service time, switching costs, and dependence between successive opportunities lead to natural extensions in which the acceptance threshold may depend on time, state, or the history of the process.

The importance of simple reference models of this kind lies in isolating the fundamental decision mechanism and determining its consequences under conditions that allow rigorous analytical results to be obtained. This makes it possible to distinguish properties resulting from the structure of the sequential choice problem itself from effects caused by additional assumptions, such as non-stationarity, dependence between observations, or variable service time. An exact solution of a simple model also provides a benchmark for evaluating more complex models, numerical algorithms, and learning procedures, and yields transparent hypotheses that can subsequently be investigated empirically.

The main motivation of the paper is to examine, within a simplified and analytically solvable model, the fundamental mechanism connecting the value of the current opportunity with the cost of further waiting and the service time following its acceptance. In the following part, we show that the resulting decision problem can be reduced to a single optimal reservation threshold. An exact solution of such a model provides a reference point for more complex extensions, threshold learning procedures, and comparisons of different sources of opportunities.

The remainder of the article is organized as follows. In Section 3, we introduce the one-armed stopping model and determine the optimal threshold rule together with the corresponding reservation threshold. Next, in Section 4, we move to the multi-armed index model and show that the selection problem reduces to comparing the reservation indices of the individual arms. In Section 5, we discuss learning the reservation threshold when the distribution of observations is unknown. In Section 6, we present an illustration of the model using the example of a bookmaker bet based on the Kelly criterion and, in Section 7, we show the connection of this example with the Kullback–Leibler divergence and the informational interpretation of the threshold rule. The article concludes with Section 8, which contains final remarks and possible directions for further research.

## 3. Optimal Stopping Rule

The idea behind the construction below refers to the works [29,30], devoted to the maximization of profit intensity. We show that the problem of an optimal investment decision can be formulated as an optimal stopping problem for an appropriately constructed stochastic process. However, the optimal rule presented here is general and does not depend on the adopted interpretation.

Consider a sequence ${\left\{X_{n}\right\}}_{n\geq 1}$ of real-valued, independent random variables with the same distribution (i.i.d.), consistent with the distribution of a random variable *X*. At successive times n=1,2,…, we observe the realization $X_{n}$ and make one of two decisions:

- **accept** (stop)—we take the current value as the reward,
- **reject** (continue)—we give up the current value and move on to the next observation.

The decision is made on the basis of the currently available information. Each observation consumes exactly 1 unit of time. When we decide to accept the value $X_{n}$, we receive a reward equal to $X_{n}$, and then incur a deterministic service time cost equal to 1 unit. Then the entire cycle renews from the beginning.

We define the length of the cycle as L:=τ+1, and the reward as $R:=X_{\tau }$. The average payoff criterion takes the form

(2) $$g\left(\tau \right):=\frac{E[R]}{E[L]}=\frac{E[X_{\tau }]}{E[\tau +1]}.$$

The aim is to maximize the above value, analogously to the Gittins problem in (1). For r∈R, a threshold rule is the stopping time

$$\tau_{r}:=\inf\left\{n\geq 1:\;X_{n}\geq r\right\}.$$

In what follows, we write $g\left(r\right):=g\left(\tau_{r}\right)$; i.e., the value of the average payoff criterion for the threshold rule with threshold *r*.

**Remark** **1.** ***Assumption on service time.*** *After the stopping time τ (acceptance), there is a deterministic service period, which we normalize to 1 unit of time. This means that the system becomes unavailable for the duration of this activity, and only after its completion is it possible to return to the observation process. This assumption models the so-called dead time or refractory period of the system, a necessary time cost associated with finalizing the decision (e.g., executing an order, physically serving a customer), during which the system cannot process new requests.*

### 3.1. Auxiliary Results

Let

(3) $${\left(X-r\right)}^{+}:=\max\left\{X-r,\;0\right\}=\left(X-r\right)\;1_{\left\{X\geq r\right\}}.$$

In particular,

$$X^{+}:={\left(X-0\right)}^{+}=\max\left\{X,0\right\}\;.$$

The quantity $E[{\left(X-r\right)}^{+}]$ is the expected excess of the variable *X* above the threshold *r*.

**Lemma** **1.** *Let $E[X^{+}]<\infty$. There exists exactly one real number $r^{⋆}$ satisfying the equation*

(4) $$E\left[{\left(X-r^{⋆}\right)}^{+}\right]-r^{⋆}=0$$

*and it is nonnegative; i.e., $r^{⋆}\in \left[0,\infty \right)$.*

**Proof.**  For r∈R, define the function

$$\phi \left(r\right):=E[{\left(X-r\right)}^{+}]-r.$$

Since

$${\left(X-r\right)}^{+}\leq X^{+}+{\left(-r\right)}^{+},$$

the assumption $E[X^{+}]<\infty$ implies that $E[{\left(X-r\right)}^{+}]<\infty$ for every *r*, and hence ϕ(r) takes finite values and ϕ(r)∈R.

For arbitrary r,s∈R, the inequality

$$|{\left(X-r\right)}^{+}-{\left(X-s\right)}^{+}|\leq |\left(X-r\right)-\left(X-s\right)|=\left|r-s\right|$$

holds. We have

$$\left|\phi \left(r\right)-\phi \left(s\right)\right|=\left|\left(E\left[{\left(X-r\right)}^{+}\right]-E\left[{\left(X-s\right)}^{+}\right]\right)-\left(r-s\right)\right|$$

and, by the triangle inequality,

$$\left|\phi \left(r\right)-\phi \left(s\right)\right|\leq \left|E\left[{\left(X-r\right)}^{+}\right]-E\left[{\left(X-s\right)}^{+}\right]\right|+|r-s|\leq \left|r-s|+|r-s\right|=2|r-s|.$$

Thus ϕ is continuous. If s>r, then ${\left(X-s\right)}^{+}\leq {\left(X-r\right)}^{+}$, and therefore

$$\phi \left(s\right)-\phi \left(r\right)=E[{\left(X-s\right)}^{+}-{\left(X-r\right)}^{+}]-\left(s-r\right)\leq -\left(s-r\right)<0,$$

so ϕ is strictly decreasing.

We now show that ϕ has a unique zero. If $E[X^{+}]=0$, then

$$\phi \left(0\right)=E\left[X^{+}\right]=0,$$

so $r^{⋆}=0$.

Assume now that $E[X^{+}]>0$. Then

$$\phi \left(0\right)=E\left[X^{+}\right]>0.$$

Moreover, for r≥0,

$$0\leq {\left(X-r\right)}^{+}\leq X^{+},$$

and hence

$$\phi \left(r\right)=E\left[{\left(X-r\right)}^{+}\right]-r\leq E\left[X^{+}\right]-r\underset{r\to +\infty }{\to }-\infty .$$

Since ϕ is continuous, the intermediate value theorem yields some $r^{⋆}>0$ such that $\phi (r^{⋆})=0$. Since ϕ is strictly decreasing, this zero is unique.

Finally, the equality $\phi (r^{⋆})=0$ is equivalent to

$$r^{⋆}=E\left[{\left(X-r^{⋆}\right)}^{+}\right],$$

so in particular $r^{⋆}\geq 0$. □

We will show that the optimization of the average payoff (2) within the class of threshold rules reduces to the maximization (with respect to the threshold *r*) of the one-dimensional function rap(r):

$$\mathrm{rap}\left(r\right):=\frac{N(r)}{1+S(r)}\;,$$

where, for r∈R,

(5) $$\begin{array}{cc} S(r) & :=P\left(X\geq r\right),\;N\left(r\right):=E\;\left[X\;1_{\left\{X\geq r\right\}}\right].\; \end{array}$$

By an equilibrium state, we mean a stationary threshold rule $\tau_{r}$ for which the value of the reservation threshold *r* agrees with the average payoff per cycle obtained by applying the same rule; that is,

rap(r)=r.

Thus, this term refers to a fixed point of the threshold mapping and not to a stationary distribution of the learning process.

**Lemma** **2.** *Let ${\left\{X_{n}\right\}}_{n\geq 1}$ be independent random variables with the same distribution as the random variable X, and let r∈R be such that S(r)>0 and $E[X^{+}]<\infty$. Then the time $\tau_{r}:=\inf\left\{n\geq 1:\;X_{n}\geq r\right\}$ is finite, $E[\tau_{r}]<\infty$, and*

$$g\left(\tau_{r}\right)=\frac{E[X_{\tau_{r}}]}{E[\tau_{r}+1]}=\frac{N(r)}{1+S(r)}=\mathrm{rap}\left(r\right).$$

**Proof.**  Set p:=S(r)∈(0,1] and q:=1−p=P(X<r)∈[0,1).

Using the assumption that the random variables $X_{i}$ are independent, for n≥0 we have

$$P\left(\tau_{r}>n\right)=P\left(X_{1}<r,\ldots ,X_{n}<r\right)=q^{n}.$$

Thus the random variable $\tau_{r}$ has the geometric distribution on {1,2,…} with parameter *p*:

$$P\left(\tau_{r}=k\right)=q^{k-1}p,\;k\geq 1.$$

In particular, $P(\tau_{r}<\infty )=1$. Using the formula for the sum of a geometric series, we obtain

(6) $$E\left[\tau_{r}\right]=\sum_{n=0}^{\infty }P\left(\tau_{r}>n\right)=\sum_{n=0}^{\infty }q^{n}=\frac{1}{p},\;E\left[\tau_{r}+1\right]=1+E\left[\tau_{r}\right]=1+\frac{1}{p}=\frac{1+p}{p}.$$

Observe that the random variable $X_{\tau_{r}}$ is integrable. Since the events $\left\{\tau_{r}=k\right\}$, k≥1, are pairwise disjoint and $P(\tau_{r}<\infty )=1$, we have

$$X_{\tau_{r}}^{+}=\sum_{k=1}^{\infty }X_{k}^{+}1_{\left\{\tau_{r}=k\right\}}\;.$$

Hence, using the monotone convergence theorem, which allows us to interchange the expectation of a nonnegative increasing limit with the limit of the expectations, we obtain

$$E\left[X_{\tau_{r}}^{+}\right]=\sum_{k=1}^{\infty }E\left[X_{k}^{+}1_{\left\{\tau_{r}=k\right\}}\right]=\sum_{k=1}^{\infty }q^{k-1}E\left[X^{+}1_{\left\{X\geq r\right\}}\right]=\frac{E[X^{+}1_{\left\{X\geq r\right\}}]}{p}<\infty .$$

By $Y^{-}:=\max\left\{-Y,0\right\}$, denote the negative part of the random variable *Y*. On the event $\left\{\tau_{r}<\infty \right\}$, we have $X_{\tau_{r}}\geq r$, and therefore

$$X_{\tau_{r}}^{-}\leq {\left(-r\right)}^{+}.$$

Hence

$$E\left[X_{\tau_{r}}^{-}\right]\leq {\left(-r\right)}^{+}<\infty .$$

Thus $E[|X_{\tau_{r}}|]<\infty$.

From the definition of $\tau_{r}$, it follows that

$$\left\{\tau_{r}=k\right\}=\left\{X_{1}<r,\ldots ,X_{k-1}<r,\;X_{k}\geq r\right\},$$

and hence

$$1_{\left\{\tau_{r}=k\right\}}=1_{\left\{X_{k}\geq r\right\}}\prod_{j=1}^{k-1}1_{\left\{X_{j}<r\right\}}.$$

Consequently,

$$E\left[X_{\tau_{r}}\right]=\sum_{k=1}^{\infty }E\left[X_{k}\;1_{\left\{X_{k}\geq r\right\}}\prod_{j=1}^{k-1}1_{\left\{X_{j}<r\right\}}\right].$$

By the independence of the variables $X_{1},\ldots ,X_{k}$ and the assumption that they are identically distributed, we obtain:

$$E\left[X_{k}\;1_{\left\{X_{k}\geq r\right\}}\prod_{j=1}^{k-1}1_{\left\{X_{j}<r\right\}}\right]=E\left[X\;1_{\left\{X\geq r\right\}}\right]\cdot {\left(P(X<r)\right)}^{k-1}=N\left(r\right)\;q^{k-1}.$$

Thus

$$E\left[X_{\tau_{r}}\right]=\sum_{k=1}^{\infty }N\left(r\right)\;q^{k-1}=\frac{N(r)}{1-q}=\frac{N(r)}{S(r)}.$$

Substituting the above into the definition (2), we get

$$g\left(\tau_{r}\right)=\frac{E[X_{\tau_{r}}]}{E[\tau_{r}+1]}=\frac{N(r)/S(r)}{\left(1+S(r))/S(r\right)}=\frac{N(r)}{1+S(r)}=\mathrm{rap}\left(r\right).$$

□

### 3.2. Optimal Reservation Threshold

The functional

(7) $$J_{\lambda }\left(r\right):=E\left[X_{\tau_{r}}-\lambda \left(\tau_{r}+1\right)\right]$$

is the standard transformation of a quotient problem into a difference form, cf. the classical Dinkelbach method for fractional programming [31,32]. In our case, for every threshold *r* such that S(r)>0, we have

(8) $$J_{\lambda }\left(r\right)=E\left[\tau_{r}+1\right]\left(\frac{E[X_{\tau_{r}}]}{E[\tau_{r}+1]}-\lambda \right)=E\left[\tau_{r}+1\right]\left(g(\tau_{r})-\lambda \right)=E\left[\tau_{r}+1\right]\left(\mathrm{rap}(r)-\lambda \right).$$

Since $E[\tau_{r}+1]>0$, the sign of $J_{\lambda }\left(r\right)$ is the same as the sign of $g\left(\tau_{r}\right)-\lambda =\mathrm{rap}\left(r\right)-\lambda$. In the proof of the theorem below, we will apply this observation for $\lambda =r^{⋆}$, where $r^{⋆}$ is the unique solution of the equation

$$E[{\left(X-r\right)}^{+}]-r=0.$$

We will show that the solution of the above equation determines the optimal reservation threshold within the class of threshold rules.

**Theorem** **1.** *Let ${\left\{X_{n}\right\}}_{n\geq 1}$ be i.i.d. with the same distribution as X, let $E[X^{+}]<\infty$, and let S(0)>0. Denote by $r^{⋆}\in \left[0,\infty \right)$ the unique solution of the equation*

$$E\left[{\left(X-r^{⋆}\right)}^{+}\right]-r^{⋆}=0.$$

*Then*

(9) $$\underset{r:\;S(r)>0}{\sup}\mathrm{rap}\left(r\right)=r^{⋆},$$

*and*

(10) $$\mathrm{rap}\left(r^{⋆}\right)=r^{⋆}.$$

*Therefore, $r^{⋆}$ determines the unique equilibrium state.*

**Proof.**  Let r∈R be such that S(r)>0. By Lemma 2, we have

$$E\left[X_{\tau_{r}}\right]=\frac{N(r)}{S(r)},\;E\left[\tau_{r}+1\right]=\frac{1+S(r)}{S(r)}.$$

Hence

$$J_{\lambda }\left(r\right)=\frac{N(r)-\lambda S(r)-\lambda }{S(r)}=\frac{E[\left(X-\lambda \right)1_{\left\{X\geq r\right\}}]-\lambda }{S(r)}.$$

Substituting $\lambda =r^{⋆}$, we obtain

$$J_{r^{⋆}}\left(r\right)=\frac{E\left[\left(X-r^{⋆}\right)1_{\left\{X\geq r\right\}}\right]-r^{⋆}}{S(r)}.$$

Since

$$\left(X-r^{⋆}\right)1_{\left\{X\geq r\right\}}\leq {\left(X-r^{⋆}\right)}^{+},$$

it follows from the definition of $r^{⋆}$ that

$$J_{r^{⋆}}\left(r\right)\leq \frac{E\left[{\left(X-r^{⋆}\right)}^{+}\right]-r^{⋆}}{S(r)}=0.$$

By (8), we therefore obtain

$$\mathrm{rap}\left(r\right)\leq r^{⋆}\;\mathrm{for}\;\mathrm{every}\;r\;\mathrm{such}\;\mathrm{that}\;S\left(r\right)>0.$$

Thus

$$\underset{r:\;S(r)>0}{\sup}\mathrm{rap}\left(r\right)\leq r^{⋆}.$$

It remains to verify that the threshold $r^{⋆}$ is admissible—i.e., $S(r^{⋆})>0$—and attains this value. If $S(r^{⋆})=0$, then ${\left(X-r^{⋆}\right)}^{+}=0$, and hence the equation

$$E\left[{\left(X-r^{⋆}\right)}^{+}\right]-r^{⋆}=0$$

would imply $r^{⋆}=0$. But then $S\left(r^{⋆}\right)=S\left(0\right)>0$, which gives a contradiction.

For $r=r^{⋆}$, we have

$$J_{r^{⋆}}\left(r^{⋆}\right)=\frac{E\left[\left(X-r^{⋆}\right)1_{\left\{X\geq r^{⋆}\right\}}\right]-r^{⋆}}{S(r^{⋆})}=\frac{E\left[{\left(X-r^{⋆}\right)}^{+}\right]-r^{⋆}}{S(r^{⋆})}=0.$$

Using (8) again, we obtain

$$\mathrm{rap}\left(r^{⋆}\right)=r^{⋆}.$$

Consequently,

$$\underset{r:\;S(r)>0}{\sup}\mathrm{rap}\left(r\right)=r^{⋆}.$$

□

**Remark** **2.** *The assumption S(0)>0 serves only to exclude the degenerate case and ensures that the threshold $r^{⋆}$ is admissible; i.e., $S(r^{⋆})>0$.*

**Remark** **3** (One-armed interpretation). *The obtained optimal threshold $r^{⋆}$ can be interpreted as the reservation value of a single source of offers. The rule*

$$\tau_{r^{⋆}}=\inf\left\{n\geq 1:\;X_{n}\geq r^{⋆}\right\}$$

*compares the current offer $X_{n}$ with the reservation threshold $r^{⋆}$. This threshold determines the boundary value at which accepting the offer and continuing to wait are equivalent. This follows from the equation*

$$E\left[{\left(X-r^{⋆}\right)}^{+}\right]=r^{⋆},$$

*which shows that the expected excess attainable as a result of further waiting is equal to the reservation threshold. The one-armed solution presented above constitutes an elementary building block for the multi-armed model, in which each arm has its own reservation value.*

It is worth emphasizing that the threshold rule associated with $r^{⋆}$ is optimal within the class of all admissible stopping times, and not only within the class of threshold rules.

By an admissible stopping time, we mean a stopping time τ with respect to the natural filtration

$$F_{n}=\sigma \left(X_{1},\ldots ,X_{n}\right),$$

taking values in {1,2,…}∪{∞} and satisfying

E[τ]<∞.

This condition already implies that

P(τ<∞)=1,

so no separate almost-sure finiteness assumption is required. The condition E[τ]<∞ is natural in the present renewal-reward setting because it guarantees a finite expected cycle length, which appears in the denominator of the average payoff criterion. Allowing stopping times with infinite expected cycle length would require a different asymptotic performance criterion and is outside the scope of the present model.

Let τ be any admissible stopping time. We first show that the positive part of the stopped payoff relative to $r^{⋆}$ is integrable. Since P(τ<∞)=1, Tonelli’s theorem gives

$$E\left[{\left(X_{\tau }-r^{⋆}\right)}^{+}\right]=\sum_{n\geq 1}E\;\left[{\left(X_{n}-r^{⋆}\right)}^{+}1_{\left\{\tau =n\right\}}\right].$$

Because

{τ=n}⊆{τ≥n},

we obtain

$$E\left[{\left(X_{\tau }-r^{⋆}\right)}^{+}\right]\leq \sum_{n\geq 1}E\;\left[{\left(X_{n}-r^{⋆}\right)}^{+}1_{\left\{\tau \geq n\right\}}\right].$$

The event {τ≥n} belongs to $F_{n-1}$ and is therefore determined before $X_{n}$ is observed. Since the observations are independent and identically distributed, the event {τ≥n} is independent of $X_{n}$, and $X_{n}$ has the same distribution as *X*. Consequently,

$$\begin{array}{cc} E[{\left(X_{\tau }-r^{⋆}\right)}^{+}] & \leq \sum_{n\geq 1}E\left[{\left(X_{n}-r^{⋆}\right)}^{+}\right]P\left(\tau \geq n\right)\\ & =E\left[{\left(X-r^{⋆}\right)}^{+}\right]\sum_{n\geq 1}P\left(\tau \geq n\right)\\ & =r^{⋆}E\left[\tau \right]<\infty , \end{array}$$

where we used the defining equation

$$E\left[{\left(X-r^{⋆}\right)}^{+}\right]=r^{⋆}$$

and the tail-sum identity

$$\sum_{n\geq 1}P\left(\tau \geq n\right)=E\left[\tau \right].$$

It follows that the positive part of $X_{\tau }-r^{⋆}$ has finite expectation. Hence $E[X_{\tau }-r^{⋆}]$ is well defined as an extended real number in [−∞,∞). In particular, neither the value +∞ nor an indeterminate expression of the form ∞−∞ can occur. Therefore,

(11) $$E\left[X_{\tau }-r^{⋆}\right]\leq E\left[{\left(X_{\tau }-r^{⋆}\right)}^{+}\right]\leq r^{⋆}E\left[\tau \right].$$

Thus,

$$E\left[X_{\tau }\right]\leq r^{⋆}\left(E[\tau ]+1\right)=r^{⋆}E\left[\tau +1\right].$$

If $E[X_{\tau }]=-\infty$, we use the convention

g(τ)=−∞.

Otherwise, the average payoff is defined in the usual way. Since

0<E[τ+1]<∞,

in both cases we obtain

$$g\left(\tau \right)=\frac{E[X_{\tau }]}{E[\tau +1]}\leq r^{⋆}.$$

Finally, consider the threshold stopping time

$$\tau_{r^{⋆}}=\inf\left\{n\geq 1:\;X_{n}\geq r^{⋆}\right\}.$$

Since $S(r^{⋆})>0$, this stopping time has a geometric distribution with success probability $S(r^{⋆})$, and therefore

$$E\left[\tau_{r^{⋆}}\right]=\frac{1}{S(r^{⋆})}<\infty .$$

Thus, $\tau_{r^{⋆}}$ is admissible. Moreover, it attains the value

$$g\left(\tau_{r^{⋆}}\right)=r^{⋆}.$$

Consequently, $\tau_{r^{⋆}}$ is optimal within the class of all admissible stopping times, and the optimal value of the stopping problem is equal to $r^{⋆}$.

The argument also covers heavy-tailed payoff distributions, provided that their positive part satisfies the standing assumption $E[X^{+}]<\infty$. The negative part of the stopped payoff may have infinite expectation, in which case the average payoff is assigned the value −∞.

**Remark** **4.** *More generally, the model may allow for a sequence of nonnegative random service times having the same distribution as a random variable Y, independent of the observation sequence, with finite mean*

μ=E[Y]∈(0,∞).

*The service time is assumed not to be known before the acceptance decision. In this case, the length of a cycle is τ+Y, and the average payoff criterion becomes*

$$g_{Y}\left(\tau \right)=\frac{E[X_{\tau }]}{E[\tau +Y]}=\frac{E[X_{\tau }]}{E[\tau ]+\mu }.$$

*For the threshold rule $\tau_{r}$, this gives*

$${\mathrm{rap}}_{\mu }\left(r\right)=\frac{N(r)}{1+\mu S(r)}.$$

*The optimal threshold $r_{\mu }^{⋆}$ is the unique nonnegative solution of*

$$r_{\mu }^{⋆}=\mu \;E\left[{\left(X-r_{\mu }^{⋆}\right)}^{+}\right],$$

*and the corresponding optimal average payoff is*

$$\underset{r:\;S(r)>0}{\sup}{\mathrm{rap}}_{\mu }\left(r\right)={\mathrm{rap}}_{\mu }\left(r_{\mu }^{⋆}\right)=\frac{r_{\mu }^{⋆}}{\mu }.$$

*Thus, the threshold structure of the solution is preserved, although for μ≠1 the optimal threshold is no longer numerically equal to the optimal average payoff. For μ=1, we recover the equations used in the main model.*

*This extension relies on the assumption that the service time is independent of the accepted payoff and is not observed before the acceptance decision. If the service time depends on the accepted opportunity or is known in advance, the optimal rule may depend on both the payoff and the service time and requires a separate analysis.*

To illustrate the dependence of the optimal reservation threshold on the reward distribution, Table 1 presents the corresponding values for five standard distributions: uniform, exponential, gamma, Weibull, and normal. The parameters of all distributions were chosen so that their expected value was equal to 1. In each case, the optimal threshold is determined by the fixed-point equation

$$E\left[{\left(X-r^{⋆}\right)}^{+}\right]=r^{⋆}.$$

Although all five distributions have the same expected reward, their corresponding optimal reservation thresholds differ. Among the cases considered, the largest threshold value is obtained for the normal distribution with variance equal to 1. This shows that the optimal threshold depends on the entire reward distribution, including its dispersion, support, and tail behavior, not only on its expected value.

## 4. Multi-Armed Index Model

The multi-armed bandit model can be interpreted as a simplified market model in which individual arms correspond to different sources of investment opportunities. An arm may be a specific asset, trading strategy, market sector, asset class, or a particular method of capital allocation. Activating a given arm leads to the observation of the next value generated by this source. This is the value of the current investment opportunity; for example, the potential net profit from a transaction, the rate of return, or a more broadly understood payoff associated with a given opportunity. Accepting this value means realizing the investment opportunity, whereas rejecting it means giving up this possibility and continuing the search. Since a rejected observation does not generate a payoff, but only lengthens the cycle time, the multi-armed problem can be interpreted as a selection process among competing market opportunities. In this interpretation, the index of each arm measures the attractiveness of a given source of investment opportunities relative to the value of further waiting.

We consider a finite set of arms

I={1,…,m}.

Each arm i∈I corresponds to an i.i.d. sequence

$$X_{1}^{\left(i\right)},X_{2}^{\left(i\right)},\ldots$$

with the same distribution as the random variable $X^{\left(i\right)}$. At a decision time, the investor selects one arm and observes one offer from this arm. After the observation, the investor may:

1. accept the offer and end the cycle with a reward equal to the observed value,
2. reject the offer and, at the next time, choose any arm again.

After acceptance, there is a deterministic service time equal to 1.

**Definition** **1.** *For a fixed arm i∈I, we define its reservation index by*

$$r_{i}^{⋆}\in \left[0,\infty \right),\;E\left[{\left(X^{\left(i\right)}-r_{i}^{⋆}\right)}^{+}\right]=r_{i}^{⋆},$$

*provided that $E[{\left(X^{\left(i\right)}\right)}^{+}]<\infty$ and $P(X^{\left(i\right)}\geq 0)>0$.*

**Remark** **5.** *By the one-armed analysis, for each arm i the number $r_{i}^{⋆}$ exists, is uniquely determined, and satisfies*

$$r_{i}^{⋆}=\underset{r:\;S_{i}\left(r\right)>0}{\sup}{\mathrm{rap}}_{i}\left(r\right),\;{\mathrm{rap}}_{i}\left(r\right):=\frac{N_{i}\left(r\right)}{1+S_{i}\left(r\right)},$$

*where*

$$S_{i}\left(r\right):=P\left(X^{\left(i\right)}\geq r\right),\;N_{i}\left(r\right):=E\left[X^{\left(i\right)}1_{\left\{X^{\left(i\right)}\geq r\right\}}\right].$$

**Definition** **2.** *Let*

$$r_{\max}:=\max_{i\in I}r_{i}^{⋆}.$$

*An arm $i^{⋆}\in I$ is called an index-optimal arm if*

$$r_{i^{⋆}}^{⋆}=r_{\max}.$$

In the described model, the reservation indices $r_{i}^{⋆}$ can be treated as values that order the arms according to their attractiveness. If, after each rejection, any arm can be freely chosen, there are no switching costs or learning effects, and the observations generated by different arms are independent, then the natural choice is to activate the arm with the largest reservation index.

For each arm *i*, we have $r_{\max}\geq r_{i}^{⋆}$, and therefore

$$E\left[{\left(X^{\left(i\right)}-r_{\max}\right)}^{+}\right]\leq r_{\max}.$$

This means that no arm gives an expected excess above the level $r_{\max}$ that would justify choosing an arm with a lower index. On the other hand, every arm $i^{⋆}$ satisfying

$$r_{i^{⋆}}^{⋆}=r_{\max}$$

attains the value $r_{\max}$ if it is continuously activated and the threshold rule

$$\tau_{r_{\max}}^{\left(i^{⋆}\right)}=\inf\left\{n\geq 1:\;X_{n}^{\left(i^{⋆}\right)}\geq r_{\max}\right\}$$

is applied to it. In this sense, the multi-armed selection problem reduces to comparing the reservation indices $r_{1}^{⋆},\ldots ,r_{m}^{⋆}$. Since no arm gives an expected excess above the level $r_{\max}$ greater than the cost of further waiting, mixing or switching between arms cannot improve the value attained by the arm with the largest index.

## 5. Learning the Reservation Threshold with an Unknown Distribution

### 5.1. General Scheme for Learning the Reservation Threshold

In the preceding considerations, we assumed that the distribution of the random variable *X* is known, which makes it possible to determine the optimal threshold $r^{⋆}$ as the solution of a fixed-point equation. In market practice, however, this assumption is too strong. The investor observes only successive realizations of investment opportunities and, on their basis, must gradually learn the value of a single arm. In the proposed model, this learning involves estimating the one-dimensional decision parameter—namely, the reservation threshold *r*—by a trial-and-adjustment method. In practice, this proceeds as follows: the investor adopts a certain threshold $r_{k}$, applies the corresponding decision rule for some time, and then checks what average payoff per cycle has been achieved in this way. If the obtained value is greater than the adopted threshold, the threshold should be increased, whereas if it is smaller, the threshold should be decreased. Repeating this procedure leads to a gradual approximation of the threshold value consistent with the observed data.

For a fixed threshold $r_{k}$, we run the threshold rule

$$\tau_{r_{k}}:=\inf\left\{n\geq 1:\;X_{n}\geq r_{k}\right\}$$

for $K_{k}$ consecutive cycles. Let

$$R_{k,1},\ldots ,R_{k,K_{k}}$$

denote the payoffs obtained in these cycles, while

$$L_{k,1},\ldots ,L_{k,K_{k}}$$

denote the corresponding cycle lengths. A natural estimator of the average payoff per cycle for the threshold $r_{k}$ is the quotient

$${\hat{g}}_{k}:=\frac{\sum_{j=1}^{K_{k}}R_{k,j}}{\sum_{j=1}^{K_{k}}L_{k,j}}.$$

The estimator ${\hat{g}}_{k}$ is a ratio estimator and, for a finite number of cycles $K_{k}$, is generally biased because the expectation of a ratio need not equal the ratio of expectations. For a fixed threshold $r_{k}$, however, successive cycles are independent and identically distributed. The strong law of large numbers therefore implies that ${\hat{g}}_{k}$ is a consistent estimator and converges to the true average payoff per cycle $g(r_{k})$ as $K_{k}\to \infty$. Under additional moment assumptions, its bias is typically of order $O(1/K_{k})$ [33].

If $K_{k}$ remains fixed, the bias need not vanish and the algorithm may converge to a value different from the true threshold $r^{⋆}$. A decreasing learning step reduces random fluctuations, but does not by itself remove persistent bias. Therefore, to obtain convergence to the true threshold, the number of cycles $K_{k}$ should be increased appropriately as learning progresses. Under the standard assumptions used in the analysis of stochastic approximation algorithms, including appropriately decreasing learning steps, control of the moments of the estimation error, stability of the iterations, and stability of the fixed point, the vanishing of the bias as $K_{k}$ increases removes one of the obstacles to convergence of the algorithm to the true threshold $r^{⋆}$ [34]. A complete proof of convergence would, however, require a detailed verification of these conditions for the procedure under consideration and lies beyond the scope of this paper.

We update the threshold according to the iteration

(12) $$r_{k+1}=r_{k}+\alpha_{k}\left({\hat{g}}_{k}-r_{k}\right),$$

where ${\left(\alpha_{k}\right)}_{k\geq 0}\subset \left(0,1\right]$ is a sequence of learning step sizes. The coefficient $\alpha_{k}$ determines how strongly the new estimate ${\hat{g}}_{k}$ affects the change of the threshold: for $\alpha_{k}=1$, we obtain the full fixed-point iteration, whereas for $0<\alpha_{k}<1$, the update has an averaged character, which may increase the stability of the procedure in stochastic settings.

The interpretation of the update (12) is direct. If

$${\hat{g}}_{k}>r_{k},$$

then the current threshold is too low relative to the estimated value of the arm, and therefore the next iteration increases the threshold. If, on the other hand,

$${\hat{g}}_{k}<r_{k},$$

then the threshold is too high and is decreased. At the fixed point, when the estimated average payoff per cycle agrees with the current threshold, the update no longer changes the value of the parameter.

A special case of (12) is the full fixed-point iteration, corresponding to the choice

$$\alpha_{k}=1,$$

for which we obtain the simple scheme

$$r_{k+1}={\hat{g}}_{k}.$$

Then each new threshold value is directly equal to the estimated average payoff per cycle obtained at the previous threshold. This scheme constitutes a natural learning procedure from data: the investor tests the current threshold, observes the payoffs and realization times generated by it, and then adjusts the decision parameter to the empirically observed quality of the strategy.

**Figure 1:**
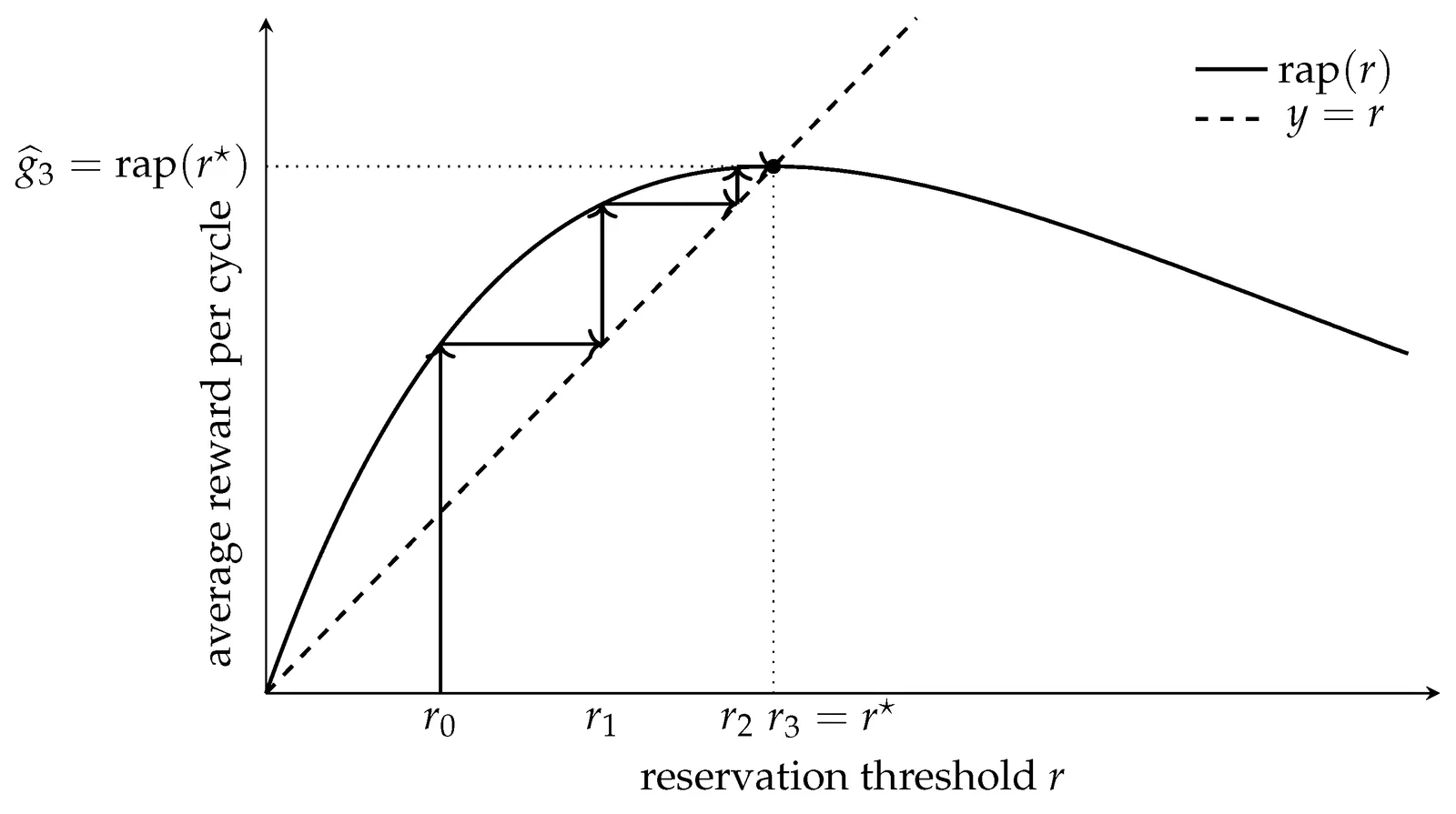
Example trajectory of learning the reservation threshold. The curve rap(r) represents the average payoff per cycle for the threshold rule with threshold *r*. Successive steps show the iteration $r_{k+1}=\mathrm{rap}\left(r_{k}\right)$, which leads to the fixed point $r^{⋆}$, satisfying $\mathrm{rap}\left(r^{⋆}\right)=r^{⋆}$. The convergence of the iteration itself requires additional regularity assumptions and does not follow solely from the existence of a fixed point.

Figure 1 presents an example trajectory of the iteration

$$r_{k+1}=\mathrm{rap}\left(r_{k}\right),$$

leading to the fixed point $r^{⋆}$. Learning in our model is considerably simpler than in general reinforcement learning [35]. Instead of approximating a value function on an extended state space, we estimate a single number, which simultaneously describes the value of the arm and generates the optimal threshold rule. The convergence of such a procedure, however, requires additional regularity conditions, in particular control of the estimation error ${\hat{g}}_{k}$. This can be done through an increasing number of observed cycles $K_{k}$ or appropriately decreasing learning step sizes $\alpha_{k}$.

### 5.2. Numerical Illustration of the Learning Process

**Figure 2:**
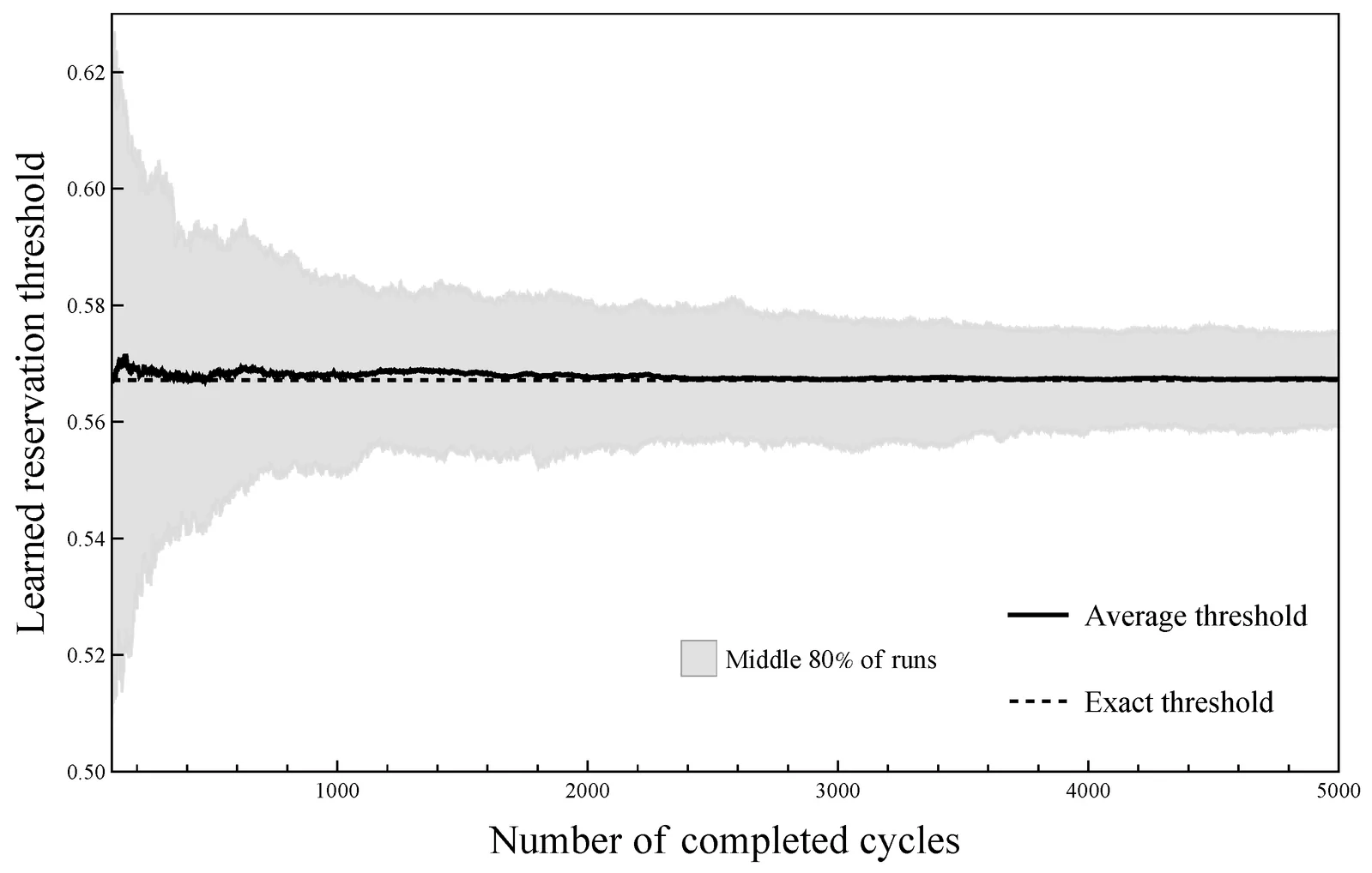
Learning the reservation threshold from rewards following an exponential distribution with rate parameter λ=1. Each of the 100 independent Monte Carlo simulations comprises 5000 completed decision cycles. The solid black line shows the mean estimated threshold in the 100 simulations. The gray region extends from the 10th to the 90th percentile and contains the middle 80% of the values obtained in the independent simulations. The horizontal dashed line indicates the exact optimal threshold $r^{⋆}=W\left(1\right)\approx 0.5671$. The narrowing of the gray region illustrates the decreasing variation in results across independent realizations of the procedure. The plot begins at cycle 100. Values for cycles 1 to 99 were omitted because of the high variability of the initial estimates.

Figure 2 presents the process of learning the reservation threshold from successively observed rewards following an exponential distribution with rate parameter λ=1. We conducted 100 independent Monte Carlo simulations, each comprising 5000 completed decision cycles. In each simulation, the initial threshold value was set to

$$r_{0}=0.$$

This is a natural choice for the exponential distribution, whose values are nonnegative. Consequently, the first reward drawn is accepted in the first cycle.

In the *k*-th cycle, the threshold $r_{k-1}$ was used. Successive reward values were drawn until a value not smaller than the current threshold appeared. This value was accepted and denoted by $R_{k}$. The cycle length $L_{k}$ was equal to the number of opportunities considered, including the accepted opportunity, increased by one additional unit of time associated with servicing the accepted opportunity.

The experiment used a fully sequential variant of the learning procedure, in which the threshold was updated after each completed cycle. Let

$$T_{k}=\sum_{j=1}^{k}L_{j}$$

denote the total duration of the first *k* cycles. After completion of the *k*-th cycle, the threshold was updated according to

$$r_{k}=\frac{\sum_{j=1}^{k}R_{j}}{T_{k}}.$$

Thus, $r_{k}$ is the ratio of the cumulative reward obtained in the first *k* cycles to the total duration of these cycles. The calculated value $r_{k}$ was then used as the threshold in cycle k+1.

The above update can also be written recursively. Since

$$\sum_{j=1}^{k-1}R_{j}=T_{k-1}r_{k-1},$$

we obtain

$$r_{k}=\frac{T_{k-1}r_{k-1}+R_{k}}{T_{k}}=r_{k-1}+\frac{L_{k}}{T_{k}}\left(\frac{R_{k}}{L_{k}}-r_{k-1}\right).$$

This update has a form analogous to the general learning scheme presented earlier. Due to the indexing adopted here, in which the *k*-th cycle is conducted using the threshold $r_{k-1}$, the corresponding quantities take the form

$$K_{k-1}=1,\;{\hat{g}}_{k-1}=\frac{R_{k}}{L_{k}},\;\alpha_{k-1}=\frac{L_{k}}{T_{k}}.$$

It is therefore a variant in which the update is performed after each individual cycle and the learning step is data-dependent. The value $\alpha_{k-1}$ is random because it depends on the length of the cycle that has just been completed.

Each of the 100 simulations generated its own sequence of estimates

$$r_{1},r_{2},\ldots ,r_{5000}.$$

Thus, for each cycle number *k*, 100 values of $r_{k}$ were obtained, one from each independent simulation. The solid black line in the figure shows the mean of these 100 values. The gray region is bounded by the 10th and 90th percentiles and therefore contains the middle 80% of the threshold values obtained in the independent simulations. This region is not a confidence interval, but a graphical illustration of the variability of the results across individual realizations of the learning procedure. Its narrowing means that, as the number of completed cycles increases, the threshold estimates obtained in different simulations become increasingly similar.

For the exponential distribution with rate parameter λ=1, the equation determining the optimal threshold takes the form

$$e^{-r^{⋆}}=r^{⋆},$$

and thus

$$r^{⋆}=W\left(1\right)\approx 0.5671,$$

where *W* denotes the Lambert function. The horizontal dashed line in the figure shows this exact value of the optimal threshold.

The experiment illustrates the empirical stabilization of the estimates near the exact value $r^{⋆}$. It does not, however, constitute a proof of convergence of the procedure used. An analytical demonstration of convergence for the variant with a random, data-dependent step $\alpha_{k}$ would require the formulation of additional regularity conditions.

## 6. An Illustrative Model of a Bookmaker’s Bet

As an illustration of the model presented in this paper, consider a sequence of horse races. Each race is interpreted as a single investment opportunity, and its value is identified with the largest attainable logarithmic gain determined by means of the Kelly criterion [36]. In the *n*-th race, the investor considers all available bets and, for each of them, determines the corresponding logarithmic capital increment in accordance with the Kelly criterion. Then $X_{n}$ is taken to be the largest of the obtained values. Consequently, the sequence ${\left(X_{n}\right)}_{n\geq 1}$ can be treated as a sequence of observations in the sense of the one-armed model presented above. Accepting the observation $X_{n}$ means placing a bet in the *n*-th race on the horse whose local Kelly value attains the maximum. This decision closes the current cycle and ties up capital for an additional unit of time, corresponding to the duration and settlement of the bet. Rejection, on the other hand, means skipping the current race and moving on to the next opportunity. As a result, the investor’s decision again takes the form of a threshold rule.

### 6.1. Single Race and Local Kelly Value

Consider the *n*-th race, in which $m_{n}$ horses take part. For each $i\in \{1,\ldots ,m_{n}\}$, denote by $p_{n,i}$ the subjective assessment of the probability that the *i*-th horse wins, and by $q_{n,i}\in \left(0,1\right)$ the share of funds bet on the *i*-th horse in the total betting pool. We assume that

$$\sum_{i=1}^{m_{n}}p_{n,i}=1\;\mathrm{and}\;\sum_{i=1}^{m_{n}}q_{n,i}=1.$$

We assume that there are no fees or taxes of any kind. This means that if the *i*-th horse wins, the unit stake placed on this event is multiplied by $1/q_{n,i}$.

To connect a single race with the binary Kelly model, for a fixed horse *i* we reduce the situation to two disjoint events:

$$A_{n,i}:=\left\{\mathrm{the}\;i-\mathrm{th}\;\mathrm{horse}\;\mathrm{wins}\right\},\;A_{n,-i}:=\left\{\mathrm{the}\;i-\mathrm{th}\;\mathrm{horse}\;\mathrm{loses}\right\}.$$

The probability of the event $A_{n,i}$ is $p_{n,i}$, and that of the event $A_{n,-i}$ is $1-p_{n,i}$. Analogously, the market share corresponding to these two events is equal to $q_{n,i}$ and $1-q_{n,i}$, respectively.

Let l∈[0,1) denote the fraction of capital staked by the player on the victory of the *i*-th horse. If this horse wins, then the player’s normalized final wealth is

$$1-l+\frac{l}{q_{n,i}}=1+\frac{1-q_{n,i}}{q_{n,i}}\;l\;.$$

In the case of a loss, it is equal to 1−l. The expected value of the logarithmic capital increment has the form

(13) $$K_{n,i}\left(l\right):=p_{n,i}\ln\;\left(1+\frac{1-q_{n,i}}{q_{n,i}}\;l\right)+\left(1-p_{n,i}\right)\ln\left(1-l\right).$$

By the optimal stake, we shall mean the quantity

(14) $$l_{n,i}^{K}:=\arg\max_{\;0\leq l<1}K_{n,i}\left(l\right).$$

If $p_{n,i}>q_{n,i}$, then the maximum is attained for

(15) $$l_{n,i}^{K}=\frac{p_{n,i}-q_{n,i}}{1-q_{n,i}},$$

whereas if $p_{n,i}\leq q_{n,i}$, the optimal stake is equal to 0. The Kelly value for the *i*-th horse in the *n*-th race is defined by

(16) $$\kappa_{n,i}:=\underset{\;0\leq l<1}{\sup}K_{n,i}\left(l\right).$$

The above quantity is a measure of the ex ante attractiveness of betting on the victory of the *i*-th horse, measured by the maximal attainable expected logarithmic capital increment in a single race. If the subjective probability that the *i*-th horse wins is greater than the probability implied by the market—i.e., $p_{n,i}>q_{n,i}$—then, according to the Kelly criterion, one should bet on its victory the fraction of capital specified by Formula (15). Otherwise, one should refrain from placing the bet. This criterion rules out betting the entire capital: when l=1, a loss reduces the final wealth to 0, and the term ln(1−l) is not defined.

Passing from a single horse to the entire race, we define its local value by

(17) $$X_{n}:=\max_{1\leq i\leq m_{n}}\kappa_{n,i}.$$

If the above maximum is attained for more than one horse, then we choose any index $i_{n}^{⋆}\in \arg\max_{1\leq i\leq m_{n}}\kappa_{n,i}$.

The i.i.d. assumption concerns the sequence of local race values $\left(X_{n}\right)$, not the individual parameters of each race. The fact that the number of horses and the subjective and market-implied probabilities vary between races is not in itself inconsistent with this assumption. These quantities may be treated as different realizations generated by the same probabilistic mechanism.

In real betting markets, these conditions may not be satisfied. Changes in the number or structure of races, the method used to determine market probabilities, the behavior of market participants, and other time-dependent factors may change the distribution of $X_{n}$ or introduce dependence between successive local values. In such cases, the theoretical results derived under the i.i.d. assumption do not apply directly. The model would then have to be extended to a non-stationary or possibly state-dependent setting in which the thresholds could depend on time, market conditions, or the observed characteristics of a race.

Accepting the observation $X_{n}$ means placing a bet in the *n*-th race on the horse $i_{n}^{⋆}$, using the stake $l_{n,i_{n}^{⋆}}^{K}$. Rejection means skipping the current race and moving on to the next opportunity. After acceptance, the capital remains committed for one further unit of time, corresponding to the duration and settlement of the bet. As a result, the sequence ${\left(X_{n}\right)}_{n\geq 1}$ can be treated as a sequence of observations in a one-armed stopping problem, and the Kelly criterion provides a concrete construction of the local value appearing in the threshold rule.

### 6.2. Reservation Threshold for Local Kelly Values

In the described model, to each race we assign a one-dimensional local value

$$X_{n}=\max_{1\leq i\leq m_{n}}\kappa_{n,i},$$

which determines the potential attractiveness of the best available opportunity in a given race. We assume here that successive races are treated as independent observations; that is, that the conditional probability distribution of future outcomes is independent of what happened in the past.

In this interpretation, the function

(18) $$\mathrm{rap}\left(a\right)=\frac{E[X\;1_{\left\{X\geq a\right\}}]}{1+P(X\geq a)}$$

describes the average logarithmic payoff per cycle obtained by a strategy that accepts only those races for which the local Kelly value satisfies $X_{n}\geq a$.

The quantity in (18) can be interpreted as the quotient of the expected value of the accepted opportunity and the expected length of the entire cycle of waiting and settlement of the bet. Maximizing rap(a) therefore expresses the trade-off between selectivity and the frequency of placing a bet. The optimal threshold $r^{⋆}$, determined by the fixed-point equation, is the reservation value of the local Kelly value. If the current race has value $X_{n}=r^{⋆}$, then the investor is indifferent between placing the best available bet in this race and continuing to wait for the next opportunity. Hence, the investor accepts the race if and only if $X_{n}\geq r^{⋆}$. After accepting a race, the investor chooses the horse $i_{n}^{⋆}\in \arg\max_{1\leq i\leq m_{n}}\kappa_{n,i}$ and bets on its victory the fraction of capital determined by the Kelly criterion. If $X_{n}<r^{⋆}$, then the race is skipped.

It is worth emphasizing that the Kelly criterion is not merely a technical rule for determining the stake, but a principle connecting capital growth with the information contained in the structure of the bet. The privileging of the strategy maximizing the expected value of the logarithm of wealth can be interpreted through projective symmetries, as well as in the context of its natural connection with Boltzmann–Shannon entropy and with constraints relevant to large investors [37]. In this sense, the optimality of the Kelly criterion goes far beyond the question “how much to stake?” It is a way of describing the relationship between information, risk, and the long-run rate of capital growth.

## 7. Kullback–Leibler Divergence and the Informational Interpretation of the Threshold Rule

In this section, we show that in the bookmaker model based on the Kelly criterion, the local value of an investment opportunity can be interpreted directly in terms of the Kullback–Leibler divergence. As a result, the reservation threshold receives an additional informational interpretation.

### 7.1. Kullback–Leibler Divergence for a Single Bet

Let *P* and *Q* be discrete probability distributions defined on the same set of values. We assume that P(x)>0 implies Q(x)>0. The Kullback–Leibler divergence of the distribution *P* relative to *Q* is defined by

$$D_{\mathrm{KL}}\left(P∥Q\right):=\sum_{x}P\left(x\right)\ln\frac{P(x)}{Q(x)},$$

where the sum runs over all values *x* for which the distributions under consideration are defined. For Bernoulli distributions Ber(p) and Ber(q), where p,q∈(0,1), the above formula takes the following form:

(19) $$D_{\mathrm{KL}}\left(\mathrm{Ber}\left(p\right)∥\mathrm{Ber}\left(q\right)\right)=p\ln\frac{p}{q}+\left(1-p\right)\ln\frac{1-p}{1-q}.$$

In the horse-racing model, for a fixed race *n* and horse *i*, we considered the function

(20) $$K_{n,i}\left(l\right)=p_{n,i}\ln\;\left(1+\frac{1-q_{n,i}}{q_{n,i}}\;l\right)+\left(1-p_{n,i}\right)\ln\left(1-l\right),\;0\leq l<1,$$

expressing the expected logarithmic capital increment. If $p_{n,i}>q_{n,i}$, then this function attains its maximum for

$$l_{n,i}^{K}=\frac{p_{n,i}-q_{n,i}}{1-q_{n,i}}.$$

Substituting the above value into (20), we obtain

$$1+\frac{1-q_{n,i}}{q_{n,i}}\;l_{n,i}^{K}=1+\frac{p_{n,i}-q_{n,i}}{q_{n,i}}=\frac{p_{n,i}}{q_{n,i}},$$

and

$$1-l_{n,i}^{K}=1-\frac{p_{n,i}-q_{n,i}}{1-q_{n,i}}=\frac{1-p_{n,i}}{1-q_{n,i}}.$$

Hence

$$\kappa_{n,i}=K_{n,i}\left(l_{n,i}^{K}\right)=p_{n,i}\ln\frac{p_{n,i}}{q_{n,i}}+\left(1-p_{n,i}\right)\ln\frac{1-p_{n,i}}{1-q_{n,i}}=D_{\mathrm{KL}}\left(\mathrm{Ber}\left(p_{n,i}\right)∥\mathrm{Ber}\left(q_{n,i}\right)\right).$$

If $p_{n,i}\leq q_{n,i}$, then the maximum of the function $K_{n,i}$ on the interval [0,1) is attained for l=0, and

$$\kappa_{n,i}=K_{n,i}\left(0\right)=0.$$

Finally,

(21) $$\kappa_{n,i}=\left\{\begin{array}{cc} D_{\mathrm{KL}}\left(\mathrm{Ber}\left(p_{n,i}\right)\;∥\;\mathrm{Ber}\left(q_{n,i}\right)\right),\; & \mathrm{if}\;p_{n,i}\geq q_{n,i},\\ 0,\; & \mathrm{if}\;p_{n,i}<q_{n,i}, \end{array}\right.$$

for every *n* and *i*.

Under the restriction to nonnegative stakes, the local Kelly value coincides with the Kullback–Leibler divergence when $p_{n,i}\geq q_{n,i}$, with both quantities equal to zero at the boundary $p_{n,i}=q_{n,i}$. If $p_{n,i}<q_{n,i}$, the restriction to nonnegative stakes makes the optimal Kelly stake, and consequently the local Kelly value, equal to zero, whereas the corresponding Kullback–Leibler divergence is strictly positive.

In other words, under the restriction to nonnegative stakes, if $p_{n,i}>q_{n,i}$, the local Kelly value is equal to the Kullback–Leibler divergence between the subjective distribution $\mathrm{Ber}(p_{n,i})$ and the market distribution $\mathrm{Ber}(q_{n,i})$. The condition $p_{n,i}>q_{n,i}$ means that the subjective probability that the *i*-th horse wins is greater than the probability implied by the market. In the player’s assessment, this horse is therefore undervalued by the market, because the market-implied probability is lower than the player’s subjective assessment of the probability of victory. In this sense, a bet on this horse’s victory has, from the player’s perspective, a positive edge relative to the market valuation. The optimal Kelly stake is positive.

The local value of the entire race takes the form

$$X_{n}=\max_{1\leq i\leq m_{n}}\kappa_{n,i}=\max_{1\leq i\leq m_{n}}\left(1_{\left\{p_{n,i}>q_{n,i}\right\}}\;D_{\mathrm{KL}}\left(\mathrm{Ber}\left(p_{n,i}\right)∥\mathrm{Ber}\left(q_{n,i}\right)\right)\right).$$

Thus $X_{n}$ is the largest exploitable informational value available in the *n*-th race within the model of nonnegative stakes. For horses satisfying $p_{n,i}>q_{n,i}$, this value is equal to the corresponding Kullback–Leibler divergence.

In the bookmaker model under consideration, the local value of a single bet $\kappa_{n,i}$ has a strict informational interpretation. It measures the exploitable divergence, under the restriction to nonnegative stakes, between the subjective assessment of the probability of victory and the probability implied by the market. The larger the value of $\kappa_{n,i}$, the greater, from the player’s perspective, the informational advantage relative to the market valuation with respect to the given horse.

### 7.2. The Threshold Rule as a Filter of Informational Advantage

In representation (21), the threshold rule

$$\tau_{r}=\inf\left\{n\geq 1:\;X_{n}\geq r\right\}\;,$$

means that the player accepts the first race in which the largest exploitable informational value exceeds the level *r*. In other words, the investor places a bet only when the current informational advantage over the market is sufficiently large.

The numerator of the function rap(r) measures the expected value of the accepted informational advantage, taking into account the probability that an opportunity exceeding the threshold *r* appears.

In the above context, the threshold rule can be interpreted as an informational filter accepting only those opportunities for which the divergence between the investor’s model and the market is sufficiently large. As a result, the threshold $r^{⋆}$ plays the role of the minimal level of informational advantage required to place a bet. Referring to the multi-armed model, to each arm *i* one can assign its own random variable $X^{\left(i\right)}$, interpreted as the local informational divergence generated by a given source of market opportunities. The corresponding reservation index $r_{i}^{⋆}$ then measures the maximal long-run informational advantage per cycle attainable from this source. Choosing the arm with the largest $r_{i}^{⋆}$ therefore means choosing the source that generates the highest effective informational advantage after taking waiting time into account.

It is worth noting that the Kullback–Leibler divergence suggests a natural extension of the horse-racing example presented above. If, in a single race, capital allocation among all horses is allowed, rather than only the choice of one of them, then the divergence

$$D_{\mathrm{KL}}\left(p_{n}∥q_{n}\right)=\sum_{i=1}^{m_{n}}p_{n,i}\ln\frac{p_{n,i}}{q_{n,i}},$$

where

$$p_{n}=\left(p_{n,1},\ldots ,p_{n,m_{n}}\right),\;q_{n}=\left(q_{n,1},\ldots ,q_{n,m_{n}}\right),$$

constitutes a natural measure of divergence between the investor’s beliefs and the valuation made by the market.

## 8. Conclusions

In this article, we presented a simple analogue of the classical Gittins index approach for a one-armed decision problem with a continuous spectrum of payoffs. Unlike the original problem, the proposed model is transparent, which means that the decision mechanism is explicit. This model depends directly on the distribution of the observed variable *X* and has an immediate interpretation as the minimal value of an opportunity that justifies its acceptance. As in the Gittins problem, a complex problem of sequential choice is reduced to comparing a single number assigned to a given source of decisions. The index therefore plays the role of a reservation value, which makes it possible to order the available alternatives and then choose the one with the greatest current attractiveness.

The main result is to show that, in a model based on independent observations and the criterion of average payoff per cycle, the optimal decision has the natural form of a threshold rule, and the corresponding reservation threshold is determined by a fixed-point equation. This provides a well-defined and stable computational objective, thereby fitting into research on the search for simple, locally computed quantities that make it possible to plan sequential decisions. This is particularly important in situations where full dynamic programming is too complex or insufficiently transparent.

The basic results of the paper rely on the assumption that successive payoffs are independent and have the same stationary distribution. Related simplifying assumptions have a long tradition in classical sequential selection models [38,39,40], which played an important role in the development of optimal stopping theory and transparent stopping rules. The significance of this assumption for the horse-racing example is discussed in Section 6. In a non-stationary environment or when successive observations are dependent, a constant reservation threshold need not be optimal, and the appropriate rule may depend on time, state, or the history of the process.

The model uses the criterion of expected average payoff per unit time and therefore does not directly account for risk aversion, payoff variability, capital constraints, or the risk of large losses. In the multi-armed version, we also assume that sources of opportunities can be freely compared and selected without switching costs, transaction costs, or liquidity constraints. Including such elements could change both the value of the index and the form of the optimal selection rule.

Another limitation is the exogenous nature of the payoff distributions. In the model, decisions made by a participant do not affect future opportunities or the behavior of other participants. The model therefore does not include competition, imitation, learning by other participants, or strategic changes in market conditions. In such situations, the value of a given source of opportunities may depend on the common state of the market and the actions of the other participants.

The learning procedure presented here reduces the problem to estimating a single threshold, but a complete analysis of its convergence requires additional conditions concerning the stability of the iterations, estimation error, and the choice of learning steps. Extensions involving dependent or non-stationary observations, switching costs, risk-sensitive criteria, and strategic interactions may lead to thresholds depending on time and state and may also require dynamic programming or numerical methods. The existence and uniqueness of the optimal rule must then be examined separately.

It is also worth noting that the description of profits from bookmaker betting mentioned in the final sections of the article has rather problematic value for a gambler betting on horse races. This follows from the fact that knowledge of the probabilities of horses’ victories is based not on statistical estimators, for which appropriate measurement data are unavailable, but on highly subjective speculations made by experts concerning the current chances of a horse in a particular race. This colorful model is nevertheless an interesting point of reference for a broad family of equivalent applications of prefix coding related to information redundancy [41,42,43,44,45,46], where data resources exist that are sufficiently rich for the required estimations. The topic indicated here, because of its breadth, requires treatment in a separate article.

## Figures and Tables

**Table 1:** Optimal reservation thresholds for selected reward distributions with expected value equal to 1. The symbols φ and Φ denote the probability density function and cumulative distribution function of the standard normal distribution, respectively.

Reward Distribution	Equation Determining $r^{⋆}$	$r^{⋆}$
Uniform distribution U(0,2)	$\frac{{\left(2-r^{⋆}\right)}^{2}}{4}=r^{⋆}$	0.535898
Exponential distribution with rate parameter 1	$e^{-r^{⋆}}=r^{⋆}$	0.567143
Gamma distribution with shape parameter 2 and rate parameter 2	$\left(r^{⋆}+1\right)e^{-2r^{⋆}}=r^{⋆}$	0.530045
Weibull distribution with shape parameter 2 and scale parameter $2/\sqrt{\pi }$	$\mathrm{erfc}\left(\frac{\sqrt{\pi }}{2}r^{⋆}\right)=r^{⋆}$	0.517005
Normal distribution with expected value 1 and variance 1, N(1,1)	$\varphi \left(r^{⋆}-1\right)+\left(1-r^{⋆}\right)\Phi \left(1-r^{⋆}\right)=r^{⋆}$	0.618425

## Data Availability

Data is contained within the article.
